# Safety Signal Generation for Sudden Sensorineural Hearing Loss Following Messenger RNA COVID-19 Vaccination: Postmarketing Surveillance Using the French Pharmacovigilance Spontaneous Reporting Database

**DOI:** 10.2196/45263

**Published:** 2023-07-14

**Authors:** Hung Thai-Van, Marie-Blanche Valnet-Rabier, Maëva Anciaux, Aude Lambert, Anaïs Maurier, Judith Cottin, Tessa Pietri, Alexandre Destère, Marlène Damin-Pernik, Fanny Perrouin, Haleh Bagheri

**Affiliations:** 1 Service d’Audiologie & d’Explorations Otoneurologiques, Hospices civils de Lyon Lyon France; 2 Département d'audiologie et d'Explorations otoneurologiques Hôpital Universitaire de lyon 1 Villeurbanne France; 3 Institut de l'Audition, Institut Pasteur Paris France; 4 Regional Pharmacovigilance Center of Franche-Comté, Centre Hospitalier Universitaire de Besançon Besançon France; 5 Regional Pharmacovigilance Center of Strasbourg, Hôpitaux Universitaires de Strasbourg, Strasbourg, France Strasbourg France; 6 Service de Pharmacosurveillance, Centre Régional de Pharmacovigilance du Centre Val de Loire, Centre Hospitalier Universitaire, Tours. France Tours France; 7 Regional Pharmacovigilance Center of Lyon, Service Hospitalo-Universitaire de Pharmaco-Toxicologie, Hospices Civils de Lyon, Lyon, France Lyon France; 8 Regional Pharmacovigilance Center of Marseille, Service de Pharmacologie Clinique et Pharmacovigilance, Aix Marseille Université, Assistance Publique Hôpitaux de Marseille, Marseille, France Marseille France; 9 Department of Pharmacology and Pharmacovigilance Center, Côte d'Azur University Medical Center, Nice, France Nice France; 10 Regional Pharmacovigilance Center of Saint-Etienne, Centre Hospitalier Universitaire de Saint-Étienne, Saint-Étienne, France Saint Etienne France; 11 Regional Pharmacovigilance Center of Nantes, Service de Pharmacologie Clinique, Nantes, France Nantes France; 12 Department of Medical and Clinical Pharmacology Regional Pharmacovigilance Center of Toulouse, CIC1436 Toulouse University Hospital Toulouse France

**Keywords:** mRNA COVID-19 vaccine, COVID-19, messenger RNA, tozinameran, elasomeran, sudden sensorineural hearing loss, audiogram, positive rechallenge, spontaneous reporting, postmarketing, surveillance, pharmacovigilance

## Abstract

**Background:**

The World Health Organization recently described sudden sensorineural hearing loss (SSNHL) as a possible adverse effect of COVID-19 vaccines. Recent discordant pharmacoepidemiologic studies invite robust clinical investigations of SSNHL after COVID-19 messenger RNA (mRNA) vaccines. This postmarketing surveillance study, overseen by French public health authorities, is the first to clinically document postvaccination SSNHL and examine the role of potential risk factors.

**Objective:**

This nationwide study aimed to assess the relationship between SSNHL and exposure to mRNA COVID-19 vaccines and estimate the reporting rate (Rr) of SSNHL after mRNA vaccination per 1 million doses (primary outcome).

**Methods:**

We performed a retrospective review of all suspected cases of SSNHL after mRNA COVID-19 vaccination spontaneously reported in France between January 2021 and February 2022 based on a comprehensive medical evaluation, including the evaluation of patient medical history, side and range of hearing loss, and hearing recovery outcomes after a minimum period of 3 months. The quantification of hearing loss and assessment of hearing recovery outcomes were performed according to a grading system modified from the Siegel criteria. A cutoff of 21 days was used for the delay onset of SSNHL. The primary outcome was estimated using the total number of doses of each vaccine administered during the study period in France as the denominator.

**Results:**

From 400 extracted cases for tozinameran and elasomeran, 345 (86.3%) spontaneous reports were selected. After reviewing complementary data, 49.6% (171/345) of documented cases of SSNHL were identified. Of these, 83% (142/171) of SSNHL cases occurred after tozinameran vaccination: Rr=1.45/1,000,000 injections; no difference for the rank of injections; complete recovery in 22.5% (32/142) of cases; median delay onset before day 21=4 days (median age 51, IQR 13-83 years); and no effects of sex. A total of 16.9% (29/171) of SSNHL cases occurred after elasomeran vaccination: Rr=1.67/1,000,000 injections; rank effect in favor of the first injection (*P*=.03); complete recovery in 24% (7/29) of cases; median delay onset before day 21=8 days (median age 47, IQR 33-81 years); and no effects of sex. Autoimmune, cardiovascular, or audiovestibular risk factors were present in approximately 29.8% (51/171) of the cases. SSNHL was more often unilateral than bilateral for both mRNA vaccines (*P*<.001 for tozinameran; *P*<.003 for elasomeran). There were 13.5% (23/142) of cases of profound hearing loss, among which 74% (17/23) did not recover a serviceable ear. A positive rechallenge was documented for 8 cases.

**Conclusions:**

SSNHL after COVID-19 mRNA vaccines are very rare adverse events that do not call into question the benefits of mRNA vaccines but deserve to be known given the potentially disabling impact of sudden deafness. Therefore, it is essential to properly characterize postinjection SSNHL, especially in the case of a positive rechallenge, to provide appropriate individualized recommendations.

## Introduction

### Background

SARS-CoV-2, the coronavirus responsible for COVID-19 disease, has spread rapidly, causing a global pandemic since early 2020. In late 2020, the first messenger RNA (mRNA)–based COVID-19 vaccine, Pfizer-BioNTech BNT162b2, Comirnaty (tozinameran), was authorized by the European Medicines Agency (EMA), followed by Moderna mRNA-1273, Spikevax (elasomeran). The monitoring of COVID-19 vaccines has been a major challenge in verifying their effectiveness in the general population and identifying any adverse drug reactions (ADRs) not yet observed in clinical trials. Postmarketing spontaneous pharmacovigilance systems provide a gold mine of data for drug safety research. Pharmacovigilance systems are designed to identify emerging signals using multiple data sources. Current pharmacovigilance is predominantly based on spontaneous reporting and mainly helpful in detecting type B effects (effects that are often allergic or idiosyncratic reactions; characteristically occur in only a minority of patients; are usually unrelated to dosage; and are serious, unexpected, and unpredictable) [1]. Signals may arise from one or multiple sources and may suggest a potentially new causal association or new aspect of a known risk of a drug. Indeed, because of the design of clinical trials, rare or delayed ADRs and those occurring in specific subpopulations could be identified only during postmarketing surveillance. Despite the use of many data sources to detect emerging signal, spontaneous reports continue to be the cornerstone of signal detection for most marketed drugs or vaccines, as they have been since the 1960s [2]. This challenge in COVID-19 vaccine surveillance seemed very important because of the large number of individuals (hundreds of thousands) exposed to these vaccines. In France, the National Agency for the Safety of Medicines and Health Products (*Agence Nationale de Sécurité du Médicament et des produits de santé* [ANSM]) is responsible for assessing the safety of vaccines through the continuous surveillance of all ADRs to quickly detect potential safety issues and take appropriate risk reduction measures. For the COVID-19 vaccination campaign, an enhanced surveillance system has been put in place [3]. If a safety signal was validated, the EMA Pharmacovigilance Risk Assessment Committee (PRAC) would make recommendations for any necessary changes to the product information or any further steps. For example, in May 2021, based on the cases reported in clinical trials and from vaccination campaigns, the EMA PRAC validated the potential signal about delayed injection site reaction for elasomeran and concluded that this information should be added to the product information of Spikevax [4]. Moreover, on October 28, 2022, the EMA PRAC recommended that heavy menstrual bleeding be added to the Summary of Product Characteristics of the mRNA vaccines (tozinameran and elasomeran) as a side effect of unknown frequency. This signal was supported by the ANSM’s requested review of all spontaneous reports in European countries via EudraVigilance—the EMA’s system for the management and analysis of information on suspected ADRs in the European Economic Area [5].

Sudden sensorineural hearing loss (SSNHL) is defined as a sensorineural hearing loss >30 dB over at least 3 consecutive audiometric frequencies occurring within <72 hours [6]. In cases where it occurs in the absence of reference audiometry, the diagnosis is made by comparing the hearing thresholds of the affected ear with those of the contralateral ear. SSNHL affects 5 to 20 individuals per 100,000 person-years [7,8], mainly those aged between 40 and 54 years [8,9]. Early detection and management of SSNHL are crucial to maximizing hearing recovery and the quality of life [10]. Although approximately 98% of SSNHL cases are unilateral [11], both their clinical presentation and prognosis vary greatly depending on the severity of the hearing loss [6], as well as the presence or absence of associated otoneurological manifestations such as tinnitus or balance disorders [6,11]. In most cases, there is no identifiable etiology, and SSNHL is classified as idiopathic [12]. The most likely pathophysiological mechanism for the development of idiopathic SSNHL is thought to be cochlear injury due to poor vascular perfusion or viral infection [13]. Disorders of the cochlear microcirculation due to altered plasma viscosity, platelet aggregability, red blood cell deformability, and endothelial dysfunction have been reported in patients with SSNHL [14,15].

According to the available literature, post–COVID-19 SSNHL is much less common than anosmia [16,17]. This may explain why post–COVID-19 SSNHL has been underdiagnosed by otolaryngologists compared with anosmia. However, several in-depth case studies have linked SSNHL cases to the COVID-19 outbreak [18-30]. The SARS-CoV-2 spike glycoprotein is known to have a particular tropism for human angiotensin-converting enzyme 2 receptors [31], which are expressed not only in the lung respiratory epithelium and the olfactory neuroepithelium but also in the vascular endothelium [32]. Accordingly, it was hypothesized that hearing loss in patients with COVID-19 might result from vascular endotheliitis with microthrombosis in the cochlea, auditory nerve, or hearing centers located in the temporal lobe [33]. Another mechanism advanced is the spread of viral meningitis from the subarachnoid space to the inner ear via the cochlear aqueduct, as supported by magnetic resonance imaging scans in clinical cases combining deafness and neurological symptoms [34]. A possible neurological manifestation of SARS-CoV-2 is the Guillain-Barré syndrome, which may induce auditory neuropathy [35]. Regardless of the pathophysiological mechanisms involved, it is interesting to note that increased high-frequency pure tone thresholds and reduced transient-evoked otoacoustic emissions, suggestive of cochlear outer hair cell damage, have been reported in asymptomatic patients who are COVID-19 positive [36].

SSNHL has recently emerged as a possible adverse effect of COVID-19 vaccines. Sporadic cases of unilateral SSNHL occurring shortly after the first or second administration of adenoviral vector– or mRNA-based vaccines have been recently reported, either isolated or in association with other cochleovestibular disorders (tinnitus, dizziness, and vertigo), in patients of both sexes with or without a history of audiovestibular, autoimmune, or cardiovascular diseases [37-40]. In February 2022, the World Health Organization (WHO) raised a signal detection about cases of SSNHL after COVID-19 vaccination, prompting the need for investigation. As of February 22, 2021, a total of 172 cases of hearing loss after COVID-19 vaccination (including 142 cases after tozinameran and 15 cases after elasomeran) were reported from 10 countries [41]. To date, 2 large-scale studies have investigated SSNHL after COVID-19 vaccine administration and reported conflicting results. One study, using data collected between December 2020 and July 2021 in the United States by the Centers for Disease Control and Prevention Vaccine Adverse Events Reporting System (a passive surveillance system), found no difference between the estimated incidence of SSNHL after COVID-19 vaccination with tozinameran, elasomeran, and ChAdOx1-S (0.6 to 28.0 cases per 100,000 person-years) and the historical incidence of SSNHL (11 to 77 cases per 100,000 person-years) [42,43]. By contrast, a retrospective cohort study conducted by the largest state-mandated health service organizations from Israel on data collected from December 2020 to May 2021 concluded that there is a proven, albeit small, risk (<1 per 100,000 vaccinated individuals) of SSNHL following immunization with tozinameran [44]. Recently, another retrospective cohort study performed on the Finish register–based countrywide data did not find a potential association between COVID-19 vaccinations and SSNHL. However, the results of these 3 studies were based on diagnosis codes in administrative databases, and the authors suggested the need for studies with more detailed clinical data with a more objective diagnosis of SSNHL [45]: none of these studies described the clinical characteristics of postvaccination SSNHL in terms of severity or duration or examined the facilitating role of potential risk factors, thereby prompting further research [46]. Moreover, the reliability of passive surveillance system databases when it comes to verifying SSNHL diagnostic criteria has been questioned [47]. To our knowledge, there is no large-scale, data-driven study investigating the cases of SSNHL after mRNA COVID-19 vaccine exposure identified using robust clinical measures of severity and recovery.

### Objectives

After several spontaneous reports of postvaccination SSNHL to the pharmacovigilance system and requests from patients and physicians for documented data regarding the possibility of revaccination (dose 2 or 3) after a first event of post–mRNA vaccine SSNHL, a national population-based active surveillance study was conducted to perform a rigorous and objective analysis of spontaneously reported cases and assess this new potential signal.

## Methods

### Study Design and Settings

The French pharmacovigilance system was established at the end of the 1970s based on a decentralized network of Regional Pharmacovigilance Centers (RPVCs), with the mission of identifying, evaluating, and preventing adverse drug events after marketing authorization. The ANSM is a decisional authority and has mobilized the French network of 30 RPVCs to ensure the continuous monitoring and evaluation of ADRs of COVID-19 vaccines through the daily evaluation of spontaneous reports. Health care professionals or users can report potential ADRs following COVID-19 vaccines (similar to how they would following the administration of other drugs) directly to the RPVC or using a dedicated web portal [48]. Each report of a potential ADR is assessed through a careful clinical, chronological, semiological, and pharmacological analysis before being registered (anonymous registration) in a centralized French Pharmacovigilance Database (FPVDB). For this purpose, an intensive pharmacovigilance survey was set up, and 6 RPVC experts were appointed to prepare weekly pharmacovigilance reports for the first marketed vaccines (4 experts for tozinameran and 2 experts for elasomeran). In addition, a scientific monitoring committee was also set up, comprising ANSM staff members, pharmacovigilance experts in charge of the survey, and specialized medical experts (cardiologists; neurologists; ear, nose, and throat specialists; etc) for a collegial evaluation of the reports submitted daily [3]. In this study, we conducted a nationwide population-based surveillance study using this enhanced pharmacovigilance system to assess a potential signal for post–mRNA COVID-19 vaccine SSNHL.

### Data Collection

We conducted a retrospective study to collect all ADRs recorded in the FPVDB occurring any time after mRNA COVID-19 vaccine administration from the beginning of the vaccination campaign (December 28, 2020) to February 2, 2022. Eligible SSNHL cases were selected using the Medical Dictionary for Regulatory Activity hierarchy, with preferred term selected from the narrow Standardized Medical Queries “Hearing impairment” [49]. The ANSM provided a line listing with all data related to ADR and the medical history generated by each RPVC. In addition, we requested medical records, audiometry examinations, magnetic resonance imaging, and all substantial examination results for each case. Two audiology experts analyzed all data according to the American Academy of Otolaryngology-Head and Neck Surgery Foundation guidelines [11]. The clinical analysis was performed on the basis of demographics (sex and age), risk factors (autoimmune, cardiovascular, or audiovestibular factors), the side of hearing loss (one or both ears affected), the presence of tinnitus or balance disorders, the degree of hearing loss (calculated by averaging hearing thresholds at 0.5, 1, 2, and 4 kHz) [50], the onset of SSNHL, and the rank of vaccination (first injection, second injection, or booster), with a particular focus on positive rechallenge cases. Following recent research on SSNHL [51,52], patients were categorized according to a grading system modified from the Siegel criteria [53]. This allowed both the quantification of hearing loss and assessment of hearing recovery outcomes using the same objective standards in all patients. The quantification of hearing loss included 5 grades covering all possible degrees of deafness: slight (grade 1), mild (grade 2), moderate to moderately severe (grade 3), severe (grade 4), and profound (grade 5). Recovery may be complete, partial, slight, or absent, or SSNHL may result in a nonserviceable ear (Tables 1 and 2).

**Table 1:** Modified Siegel criteria for hearing loss (HL) grades.

Level of HL	HL range (dB)
Grade 1	≤25
Grade 2	26-40
Grade 3	41-70
Grade 4	71-90
Grade 5	>90

**Table 2:** Modified Siegel criteria for hearing recovery outcomes.

Level of hearing recovery	Final hearing level (dB), gain (dB)
Complete recovery	≤25
Partial recovery	26-40, >15
Slight improvement	41-70, >15
No improvement	71-90, <15
Nonserviceable ear	>90

We classified the cases as medically or nonmedically documented. After a detailed review of complementary medical data, we excluded cases for which the attributable causes were different from vaccination or those that were nonevaluable because of missing data (nonmedically documented). Finally, the cases fulfilling the criteria of SSNHL were included and distinguished using a cutoff of 21 days. This cutoff value was recommended by the Safety Platform for Emergency Vaccines and the Brighton Collaboration guidelines for sensorineural hearing loss [54]. According to previous research on the auditory [43,44] or nonauditory [55] adverse events of mRNA vaccination, exposure could be defined as vaccination with an mRNA COVID-19 vaccine within the previous 21 days, and patients vaccinated beyond that date were considered unexposed. However, given the very limited number of large-scale studies available on the auditory adverse effects of mRNA COVID-19 vaccination, we also considered the spontaneous reporting of SSNHL after day 21 in the first instance.

A minimum of 3 months of follow-up was conducted (follow-up until August 1) for each SSNHL case to collect missing data and assess the potential hearing loss recovery.

### Outcome Measure

Descriptive analysis was performed for the 2 mRNA COVID-19 vaccines (tozinameran and elasomeran) for all the items cited earlier. The primary outcome was the reporting rate (Rr) of SSNHL following mRNA COVID-19 vaccine administration per 1 million doses, which was calculated using the total number of mRNA COVID-19 vaccine doses administered over the study period as the denominator according to the month of administration. Since the beginning of the French vaccination campaign against COVID-19 to early February 2022, a total of 97,840,529 doses of tozinameran and 22,690,889 doses of elasomeran have been administered in France [56].

### Statistical Analysis

Patient characteristics were expressed as count (percentage) for categorical variables and median (IQR) for quantitative variables according to the administered vaccine and for the whole cohort because of the few elasomeran cases. The Rr was expressed as cases per 1 million vaccine injections. The number of reported SSNHL cases and the number of vaccine injections were estimated by month during the study period. Finally, we performed a binomial test comparing a sample proportion with a hypothetical proportion to analyze the potential association of the Rr of SSNHL with sex, vaccination rank, and unilaterality or bilaterality of hearing loss (significance level: *P*<.05; R [version 4.2.1; R Foundation for Statistical Computing]).

### Ethics Approval

This nationwide postmarketing surveillance study was conducted with the authorization of the *Commission nationale de l’informatique et des libertés* (CNIL; National Commission on Informatics and Liberty; n 2014-302). The authorization was granted specifically for the French pharmacovigilance spontaneous reporting database placed under the responsibility of the ANSM.

## Results

**Figure 1:**
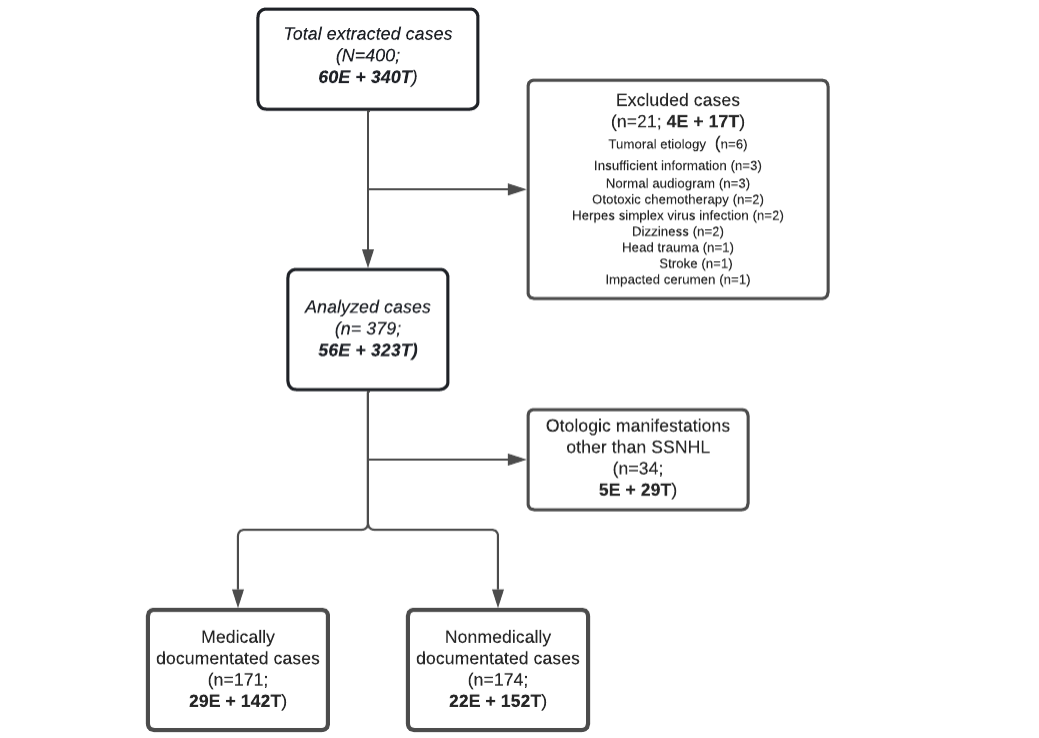
Flowchart for selection of cases of sudden sensorineural hearing loss (SSNHL) following messenger RNA (mRNA) vaccines. E: elasomeran; T: tozinameran.

During the study period, 400 cases of SSNHL after COVID-19 vaccine administration (n=340, 85% for tozinameran and n=60, 15% for elasomeran) were registered in the FPVDB. After case review, a total of 42.8% (171/400) of cases fulfilled the criteria of SSNHL (Figure 1). Hearing assessment was available either through an initial audiogram test (118/171, 69% of cases) or through an expert medical evaluation performed at the follow-up (53/171, 30.9% of cases).

### Description of Post–Tozinameran Vaccine SSNHL Cases

A total of 323 cases of SSNHL after tozinameran vaccine administration were selected, of which 142 (43.9%) cases met the criteria for inclusion; 29 (8.9%) cases were consistent with otologic manifestations other than SSNHL, and 152 (47.1%) cases were not sufficiently documented to draw conclusions (Figure 1).

For these 142 cases, the delay onset was ≤21 days for 108 (76.1%) cases, with a median delay onset of 4 (range 2-9) days. Of these 142 cases, 84 (59.2%) were those of female patients. The median age was 51 (range 13-83) years, and 69% (98/142) of patients were aged 30 to 64 years. A total of 35.2% (50/142) of patients had a medical history, which was otoneurological in 11.9% (17/142) of cases. The vaccination rank was known for 88% (125/142) of cases, with the first injection involved in 42.3% (60/142) of cases. Steroids were administered orally in 47.2% (67/142) of cases. SSNHL was unilateral in 87.3% (124/142) of cases. The hearing loss grade ranged between 1 and 3 in 75.4% (107/142) of cases. Tinnitus was associated with SSNHL in 52.8% (75/142) of cases and vertigo in 28.9% (41/142) of cases. Complete recovery was observed in 22.5% (32/142) of cases, whereas no improvement was observed in 53.5% (76/142) of cases and nonserviceable ear in 8.5% (12/142) of cases (Tables 3 and 4).

Deafness was more often unilateral than bilateral (*P*<.001). No effect of sex or vaccination rank was observed. Case follow-up identified 3.5% (5/142) of cases of positive rechallenge (Table 5): four were those of female vaccine recipients and 1 was that of a male vaccine recipient (age range 32-72 years). SSNHL was unilateral in most cases, was of grades 2 (37/142, 26.1%) to 3 (46/142, 32.4%), occurred rapidly (<10 days) after the first and second doses in all cases, and completely resolved in 2.1% (3/142) of cases during the 3-month follow-up.

**Table 3:** Characteristics of sudden sensorineural hearing loss cases.

Characteristics			Tozinameran							Elasomeran				
			<21 days (n=108, 76.1%)		>21 days (n=34, 23.9%)		Total (n=142)		<21 days (n=26, 86.7%)			>21 days (n=3, 10.3%)		Total (n=29)
**Sex, n (%)**														
	Male	43 (39.8)		15 (44.1)		58 (40.8)		13 (50)			1 (33.3)		14 (48.3)	
	Female	65 (60.2)		19 (55.9)		84 (59.2)		13 (50)			2 (66.7)		15 (51.7)	
**Age (years)**														
	Values, median (range)	50 (13-83)		51 (16-72)		51 (13-83)		51 (33-81)			43 (36-68)		47 (33-81)	
	0-18, n (%)	3 (2.8)		1 (2.9)		4 (2.8)		0 (0)			0 (0)		0 (0)	
	19-29, n (%)	7 (6.5)		0 (0)		7 (4.9)		0 (0)			0 (0)		0 (0)	
	30-49, n (%)	43 (39.8)		12 (35.3)		55 (38.7)		10 (38.5)			2 (66.7)		12 (41.4)	
	50-64, n (%)	26 (24.1)		17 (50)		43 (30.3)		9 (34.6)			0 (0)		9 (31)	
	65-74, n (%)	19 (17.6)		4 (11.8)		23 (16.2)		6 (23.1)			1 (33.3)		7 (24.1)	
	≥75, n (%)	10 (9.3)		0 (0)		10 (7)		1 (3.8)			0 (0)		1 (3.4)	
**Medical history, n (%)**			38 (35.2)		12 (35.3)		50 (35.2)		7 (26.9)			2 (66.7)		9 (31)
	CV^a^	9 (8.3)		5 (14.7)		14 (9.9)		2 (7.7)			1 (33.3)		3 (10.3)	
	ON^b^	12 (11.1)		5 (14.7)		17 (12)		3 (11.5)			1 (33.3)		4 (13.8)	
	AIM^c^	7 (6.5)		2 (5.9)		9 (6.3)		0 (0)			0 (0)		0 (0)	
	CV and ON	4 (3.7)		0 (0)		4 (2.8)		0 (0)			0 (0)		0 (0)	
	ON and AIM	3 (2.8)		0 (0)		3 (2.1)		1 (3.8)			0 (0)		1 (3.4)	
	Other etiologies	3 (2.8)		0		3 (2.1)		1 (3.8)			0 (0)		1 (3.4)	
Time to onset^d^ (days), median (IQR)			4 (2-9)		41 (25-67)		N/A^e^		8 (1-21)			50 (26-144)		N/A
**Vaccination rank, n (%)**														
	First dose	47 (43.5)		13 (38.2)		60 (42.2)		12 (46.2)			1 (33.3)		13 (44.8)	
	Second dose	39 (36.1)		14 (41.2)		53 (37.3)		10 (38.5)			2 (66.7)		12 (41.4)	
	Booster	12 (11.1)		0 (0)		12 (8.5)		2 (7.7)			0 (0)		2 (6.9)	
	Unknown	10 (9.3)		7 (20.6)		17 (12)		2 (7.7)			0 (0)		2 (6.9)	
Oral steroid administration, n (%)			48 (44.4)		19 (55.9)		67 (47.2)		14 (53.8)			2 (66.7)		16 (55.2)
**Laterality, n (%)**														
	Unilateral	94 (87)		30 (88.2)		124 (87.3)		20 (76.9)			2 (66.7)		22 (75.9)	
	Bilateral	14 (13)		4 (11.8)		18 (12.7)		6 (23.1)			1 (33.3)		7 (24.1)	
**Hearing loss grades, n (%)**														
	Grade 1	22 (20.4)		2 (5.9)		24 (16.9)		4 (15.4)			3 (100)		7 (24.1)	
	Grade 2	27 (25)		10 (29.4)		37 (26.1)		5 (19.2)			0 (0)		5 (17.2)	
	Grade 3	35 (32.4)		11 (32.4)		46 (32.4)		7 (26.9)			0 (0)		7 (24.1)	
	Grade 4	13 (12)		6 (17.6)		19 (13.4)		3 (11.5)			0 (0)		3 (10.3)	
	Grade 5	11 (10.2)		5 (14.7)		16 (11.3)		7 (26.9)			0 (0)		7 (24.1)	
**Associated cochleovestibular disorders, n (%)**														
	Tinnitus	59 (54.6)		16 (47.1)		75 (52.8)		8 (30.8)			2 (66.7)		10 (34.5)	
	Vertigo and balance disorders	33 (30.6)		8 (23.5)		41 (28.9)		11 (42.3)			1 (33.3)		12 (41.4)	
Time to recovery (days), median (range)			15 (5-67.5)		11 (8-22.5)		N/A		21 (2-90)			ND^f^		N/A
Positive rechallenge, n (%)			5 (4.6)		0 (0)		5 (3.5)		2 (7.7)			1 (33.3)		3 (10.3)

^a^CV: cardiovascular.

^b^ON: otoneurological.

^c^AIM: autoimmune disease.

^d^Interval between vaccine administration and symptom onset.

^e^N/A: not applicable.

^f^ND: not determined.

**Table 4:** Hearing recovery outcomes by grade of hearing loss.

Hearing loss	Hearing outcome										
	Tozinameran (n=142), n (%)						Elasomeran (n=29), n (%)				
	Complete recovery	Partial recovery	Slight improvement	No improvement	Nonserviceable ear	Complete recovery		Partial recovery	Slight improvement	No improvement	Nonserviceable ear
Grade 1	12 (8.5)	0 (0)	0 (0)	12 (8)	0 (0)	4 (13.8)		0 (0)	0 (0)	3 (10.3)	0 (0)
Grade 2	9 (6.3)	5 (3.5)	0 (0)	23 (16)	0 (0)	2 (6.9)		1 (3.4)	0 (0)	2 (6.9)	0 (0)
Grade 3	10 (7.1)	6 (4.2)	1 (0.7)	29 (20)	0 (0)	1 (3.4)		2 (6.9)	0 (0)	4 (13.8)	0 (0)
Grade 4	1 (0.7)	3 (2.1)	3 (2.1)	12 (8)	0 (0)	0 (0)		1 (3.4)	0 (0)	2 (6.9)	0 (0)
Grade 5	0 (0)	0 (0)	4 (2.8)	0	12 (8.5)	0 (0)		2 (6.9)	0 (0)	0 (0)	5 (17.2)

**Table 5:** Characteristics of the positive rechallenge cases.

Sex			Age (years)		Medical history		Vertigo		Tinnitus		Vaccination rank and delay onset (days)		Hearing loss side		Hearing loss grade^a^		Final hearing recovery
**Tozinameran**																	
	Female	61		None		Yes		Yes		D1^b^—4 D2^c^—7 B^d^—7		Unilateral		3		No improvement	
	Male	47		None		No		Yes		D1—9 D2—9		Unilateral		3		No improvement	
	Female	74		Fluctuating hearing loss and vertigo punctually treated with steroids		No		Yes		D1—8 D2—9		Unilateral		3		Complete recovery	
	Female	32		Protein S deficiency		No		No		D1—8 D2—2		Unilateral		2		Complete recovery	
	Female	79		Diabetes mellitus stabilized since 1977, left Ménière’ disease since 1977, hypothyroidism stabilized, high blood pressure stabilized since 1985, and hypercholesterolemia stabilized since 1985		Yes		Yes		D1—8 D2—8 B—R-^e^		Unilateral (contralateral to the Ménière side)		2		Complete recovery	
**Elasomeran**																	
	Female	45		None		No		Yes		D1—5 D2—<1 B—R-^e^		Bilateral		2		Complete recovery	
	Male	71		None		No		Yes		D1—1 D2—1		Unilateral		2		Complete recovery	
	Female	41		High blood pressure		No		Yes		D2—60 B—40		Bilateral		2		No improvement	

^a^Measured after injection.

^b^D1: first vaccine injection.

^c^D2: second vaccine injection.

^d^B: booster.

^e^R-: Negative rechallenge (no recurrence of sudden sensorineural hearing loss; the booster of elasomeran was administered at half dose).

### Description of Post–Elasomeran Vaccine SSNHL Cases

A total of 56 cases of SSNHL after elasomeran vaccine administration were selected, of which 29 (49%) cases met the inclusion criteria; 5 (8%) cases were consistent with otologic manifestations other than SSNHL, and 22 (37%) cases were not sufficiently documented to draw conclusions (Figure 1).

Among these 29 cases, the delay onset was ≤21 days for 26 (90%) cases, with a median delay onset of 8 (range 1-21) days, and 15 (52%) were those of female patients. The median age was 47 (range 33-81) years, and 72% (21/29) of patients were aged 30 to 64 years. A total of 31% (9/29) of patients had a medical history, which was otoneurological in 14% (4/29) of cases. The vaccination rank was known for 27 cases, with the first injection involved in 45% (13/29) of cases. Steroids were administered orally in 55% (16/29) of cases. SSNHL was unilateral in 76% (22/29) of cases. The hearing loss grade ranged from 1 to 3 in 66% (19/29) of cases. Tinnitus was associated with SSNHL in 34% (10/29) of cases and vertigo in 41% (12/29) of cases. Complete recovery was observed in 24% (7/29) of cases, whereas no improvement was observed in 38% (11/29) of cases and nonserviceable ear in 18% (5/29) of cases (Tables 3 and 4).

Deafness was more often unilateral than bilateral (*P*<.003). A rank effect was found in favor of the first dose (*P*<.03). No effects of sex or side of hearing loss were observed. Case follow-up identified 3 cases of positive rechallenge (Table 5): two (67%) were those of female vaccine recipients and 1 (3%) was that of a male vaccine recipient (age range 41-71 years). SSNHL was unilateral in most cases, was of grades 2 (5/29, 18%) to 3 (7/29, 24%), occurred rapidly (<10 days) after the first and second doses in 2 cases (in the third case, delay onset was >21 days, but with positive rechallenge with the booster), and completely resolved in 2 cases during the 3-month follow- up.

### Rate of Spontaneous Reports

**Figure 2:**
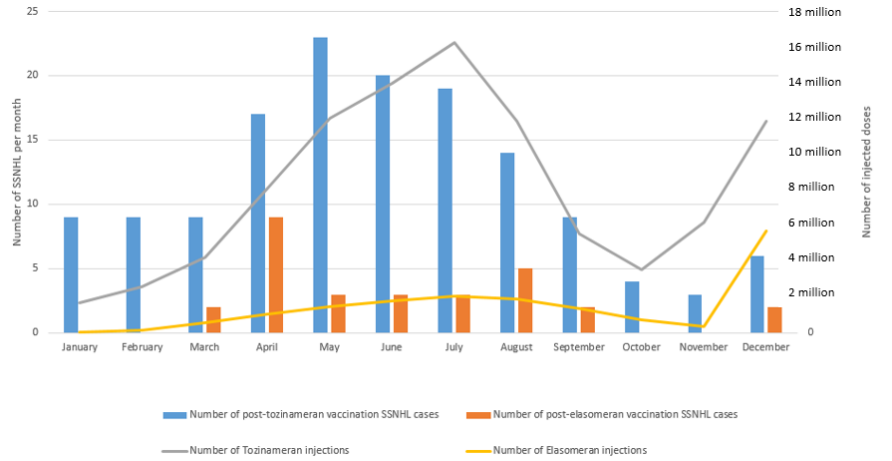
Reporting rate of sudden sensorineural hearing loss (SSNHL) cases for the 2 messenger RNA (mRNA) vaccines according to the month. Solid lines: the number of tozinameran (gray) and elasomeran (yellow) injections expressed in million doses (right y-axis) per month. Bar graph: the number of tozinameran-related (blue) and elasomeran-related (orange) SSNHL cases expressed in case number (left y-axis) per month.

Figure 2 shows, by month, the number of spontaneous reports of SSNHL cases after tozinameran vaccine administration and after elasomeran vaccine administration, as well as the number of injections of each vaccine in 2021. The total Rr seemed similar for both vaccines and was estimated at 1.45/1,000,000 doses for tozinameran and 1.67/1,000,000 doses for elasomeran. During this period, a total of 86,993 and 20,265 spontaneous reports were registered for tozinameran and elasomeran, respectively, in the FPVDB, of which 22,619 (26%) and 3782 (18.66%) were serious cases, respectively. As a result, SSNHL cases accounted for 0.63% and 0.76% of the total serious adverse events recorded for tozinameran and elasomeran, respectively.

## Discussion

### Principal Findings

The main objective of this nationwide postmarketing surveillance study was to conduct a continuous assessment of the safety of mRNA COVID-19 vaccines to generate potential safety signals related to serious and typical adverse effects and consider relevant measures that should be taken quickly [3]. Our results—based on all spontaneous reporting of mRNA vaccine–induced SSNHL registered in the FPVDB during >1 year, supported by intensive pharmacovigilance monitoring, and combined with administrative data on the doses of vaccines administered—suggested that the occurrence of SSNHL after mRNA COVID-19 vaccination could be defined as a very rare adverse effect, as it was estimated to be <2 per 1 million injections for both vaccines. The occurrence of SSNHL cases was found to parallel the number of injections of the most widely administered mRNA vaccine in France over the study period, with tozinameran accounting for 80% of the injections. This has not been verified for elasomeran injections, probably because of the smaller number of people exposed to this vaccine and the smaller number of SSNHL cases reported. The scarcity of this adverse effect supports the appropriateness of spontaneous reporting allowing a signal generation. Spontaneous reporting is particularly useful for identifying rare and idiosyncratic safety concerns. For example, 12 (57%) out of 21 drug withdrawals in France between 1998 and 2004 were solely based on spontaneous reports. Similarly, 8 (73%) out of 11 product withdrawals between 1999 and 2001 in the United Kingdom and the United States were based on early information from spontaneous reports. Some studies suggest that spontaneous reports are most useful for newly marketed products, specifically within 3 years after launch [2]. A larger investigation and disproportionality analysis of the spontaneous reporting of WHO database (Vigibase) could be useful to generate a signal. Moreover, a multicentric case-control study, methodologically appropriate to investigate rare events, might be of interest to conclude an association between mRNA COVID-19 vaccination and SSNHL, considering the different risk factors for SSNHL, as it has already been done for myocarditis or pericarditis [57,58]. That said, the Rr of mRNA COVID-19 vaccine–induced SSNHL cases remained below the estimated incidence of SSNHL cases in the general population (approximately 5 to 20 cases per 100,000 person-years in France). In addition, unlike cases of mRNA COVID-19 vaccine–induced myocarditis [58] and because of the lack of data, it should be difficult to compare the rate of SSNHL cases related to mRNA COVID-19 vaccine administration with that presumably related to COVID-19 infections.

Another contribution of this study is the first clinical description of SSNHL as an adverse effect of mRNA COVID-19 vaccination. Because strict diagnostic criteria were used, the risk of false-positive results when identifying SSNHL cases was reduced compared with previous research using passive surveillance system databases [47]. To name a few, conductive or posttraumatic hearing loss, vestibular neuritis, labyrinthitis, and fluctuating hearing loss related to a history of Ménière disease or acoustic neurinoma were excluded. Nevertheless, the clinical presentation of mRNA COVID-19 vaccine–induced SSNHL cases could include audiovestibular symptoms such as tinnitus, vertigo, or balance disorders associated with deafness. Although postvaccination hearing loss was most often found to be unilateral and of grades 1 to 3 (ie, slight to moderately severe hearing loss), there were also cases of bilateral and severe to profound hearing loss that may have required specific treatment in the absence of complete recovery for both vaccines studied. It is interesting to note that at the end of the follow-up period (minimal duration: 3 months), a complete recovery was achieved in less than one-quarter of the cases. Whereas SSNHL secondary to vaccination was found to be a very rare adverse event, it is of note that 10% of affected patients were diagnosed with a nonserviceable ear, which represents a potential indication for cochlear implantation. The rate of complete or partial recovery varied between 41% and 52% for hearing loss grades 1 to 3, whereas the rate of complete or partial recovery was 23% for grade 4 and only 9% for grade 5. This observation is in agreement with the evolution of SSNHL reported outside the vaccination context [51].

However, the rate of recovery could be greater because of missing feedback from patients who recovered completely. The most affected age group was intermediate (30-64 years), and the median age was approximately 50 years for both vaccines, which is consistent with the usual age of onset of idiopathic SSNHL [9,10]. Thus, there was no evidence for a more frequent occurrence of this adverse effect for the extreme age groups. Autoimmune, cardiovascular, audiological, or otoneurological risk factors were present in only one-third of the cases, definitively preventing the identification of a specific patient profile.

Furthermore, we should underline our findings related to the delay onset of SSNHL according to the vaccination rank. A rank effect was found only for elasomeran: the occurrence of SSNHL was higher after the first injection than after the second injection and the booster. In agreement with Safety Platform for Emergency Vaccines and Brighton Collaboration guidelines on postvaccinal SSNHL, as well as with previous data on audiological or cardiac adverse events with mRNA COVID-19 vaccines, we found that the median delay onset fell within the first 2 weeks after injection [42,43,54,57,58]. Wichova et al [39] also reported an average delay onset of 10.18 (SD 9) days, whereas the median (range) delay onset in the WHO report was 1 day (few hours to 19 days) [41].

Moreover, data supported a possible relationship between exposure to mRNA COVID-19 vaccines and the occurrence of SSNHL. Positive rechallenge cases were documented for 5 tozinameran and 3 elasomeran vaccinations, meaning that repeated exposure to a COVID-19 mRNA-based vaccine led to SSNHL for a minority of patients who had previously experienced the same adverse event. In a pharmacovigilance study, this result alone was sufficient to suggest a high probability of a causal relationship [59].

Since the beginning of the COVID-19 outbreak, the association between SARS-CoV-2 infection and the occurrence of SSNHL has been supported exclusively by cross-sectional studies conducted on limited case series [60]. Almost all reported cases were unilateral sudden deafness [26,28], which could be associated with disabling ipsilateral tinnitus [16,24] or vertigo with nystagmus beating toward the contralateral side [28]. Regardless of the clinical presentation, the estimation of the exact incidence of SSNHL attributable to COVID-19 infection may have been hampered by the difficulty in applying the same criteria for causal relationship across studies. These criteria could include, in addition to the certainty of viral infection by polymerase chain reaction test or the titration of specific antibodies, the temporal concordance between the infection and the onset of hearing loss; the presence of symptoms and signs consistent with concomitant labyrinthitis or neuritis; and, above all, the exclusion of any other reason that could be counted among the causes of SSNHL [61]. Recent updates have pointed out the need for the long-term documentation of audiovestibular symptoms in large cohorts of SARS-CoV-2–infected patients before conclusions can be drawn about their true incidence [62-64]. On the basis of the existing literature, SSNHL can be considered so far as a rare complication of COVID-19 infection [21,65,66].

### Limitations

Our study has some limitations. An important step in assessing vaccine-related adverse events is to compare their rates in vaccinated and unvaccinated populations, as was done for myocarditis [58]. Because the studied adverse event is very rare, large population-based pharmacovigilance studies (Vigibase) or a case-control study appropriate for rare events is needed to further define the role of mRNA COVID-19 vaccination in the occurrence of SSNHL, as has been done for COVID-19 vaccines and menstrual cycle [67]. Herein, we conducted a “case series study,” a well-known design in pharmacoepidemiology that differs from a cohort study or a case-control study. As such, our nationwide postmarketing surveillance did not include any control group, thus preventing the consideration of potentially important variables such as age or SSNHL time to onset. After drug marketing, case series are most useful for 2 related purposes: first, to quantify the incidence or rate of an adverse reaction and second, to detect any particular adverse effect not yet identified in clinical trials because of its scarcity [68]. Moreover, we cannot rule out the underreporting of ADRs, which may have biased the estimated rate of SSNHL. However, following the introduction of mRNA COVID-19 vaccines in France, a major effort has been made by the enhanced pharmacovigilance system to improve the reporting of any suspected ADR following the administration of these vaccines by health professionals or patients. By February 2022, approximately 80% of the French population had received the first 2 vaccine injections, and 52.6% had also received the booster [69]. The impressive number of reports of suspected ADRs (>80,000 between January 2021 and January 2022 in France) suggests that underreporting may have been very rare, particularly for potentially serious adverse events [3].

Other authors have retrospectively compared the incidence of SSNHL cases in the population exposed to mRNA COVID-19 vaccines with that in the general population using a population-based cohort and International Classification of Diseases, Ninth Revision, codes without any data on patients’ medical history or risk factors [38,39]. In our clinical experience, this could lead to a selection of false-positive SSNHL cases. As previously done by Ouldali et al [70] for mRNA COVID-19 vaccine–induced pediatric inflammatory multisystem syndrome, we chose a restrictive method to select and describe medically confirmed SSNHL cases based on pharmacovigilance and complementary medical data, which is the added value of our survey. Nevertheless, the lack of available data for the general population precluded comparisons with historical incidence or incidence in the unvaccinated population. Another possible limitation is the underdiagnosis of SSNHL cases related to the COVID-19 outbreak due to the difficulties in accessing health care during the COVID-19 outbreak. However, disabling deafness remains a medical emergency and should lead to hospital admission.

### Conclusions

This study identified very rare but severe cases of medically confirmed SSNHL after the administration of mRNA COVID-19 vaccines without calling into question the demonstrated benefit of vaccinating a large population in the context of rapid SARS-CoV-2 circulation. On the basis of our findings, particularly those related to the cases of positive rechallenge and grade-5 hearing loss, patients should undergo a comprehensive evaluation of any otologic manifestation occurring after COVID-19 vaccination. Early diagnosis of SSNHL is essential for positive outcomes. Each medical evaluation should be performed for 2 reasons: the prompt initiation of appropriate treatment to improve the prognosis of SSNHL and provision of expert counseling to patients regarding subsequent vaccine injections, given the evolution of SARS-CoV-2, its variants, and new adaptive vaccines. These data are new and of critical interest to patients, physicians, and regulatory policies to tailor public health messages and medical care.

## Abbreviations

ADR: adverse drug reaction
ANSM: Agence Nationale de Sécurité du Médicament et des Produits de santé (National Agency for the Safety of Medicines and Health Products)
CNIL: Commission nationale de l’informatique et des libertés (National Commission on Informatics and Liberty)
EMA: European Medicines Agency
FPVDB: French Pharmacovigilance Database
mRNA: messenger RNA
PRAC: Pharmacovigilance Risk Assessment Committee
RPVC: Regional Pharmacovigilance Center
Rr: reporting rate
SSNHL: sudden sensorineural hearing loss
WHO: World Health Organization

## Data Availability

Data are available upon reasonable request to MBV-R and HB.
